# The Influence of Anthropometric Variables on the Performance of Elite Traditional Rowers

**DOI:** 10.3390/sports12070185

**Published:** 2024-07-05

**Authors:** Arkaitz Castañeda-Babarro, Patxi León-Guereño, Aitor Viribay, Borja Gutiérrez-Santamaría, Iker López, Juan Mielgo-Ayuso

**Affiliations:** 1 Health, Physical Activity and Sports Science Laboratory, Department of Physical Activity and Sports, Faculty of Education and Sport, University of Deusto, 48007 Bilbao, Spain; arkaitz.castaneda@deusto.es; 2 Institute of Biomedicine (IBIOMED), University of Leon, 24004 León, Spain; aitor@glut4science.com; 3 Department of Physical Activity and Sports Sciences, Faculty of Health Sciences, Euneiz University, Vitoria-Gasteiz, Alava, La Biosfera Ibilbidea, 6, 01013 Gasteiz, Spain; borja.gutierrez@euneiz.com; 4 Kirolene, San Ignacio Auzunea Etxetaldea 5, 48200 Durango, Spain; coordinadorfc@kirolene.net; 5 Department of Health Sciences, Faculty of Health Sciences, University of Burgos, 09001 Burgos, Spain; jfmielgo@ubu.es

**Keywords:** traditional rowing, elite, body composition, anthropometry, ergometer performance

## Abstract

Athletes’ anthropometry, and especially their body composition, plays an important role in sport performance in general and in *Trainera* rowing in particular. Rowers’ anthropometric and performance profiles may vary according to their position in the boat. The objectives of this study were to investigate the relationship between anthropometry, physical performance, physiological variables, and elite male rowers’ boat positions. Twenty elite male traditional rowers were assessed and categorized according to their boat position: either in the middle of the boat (M) (*n* = 9) or in the bow and stern positions (BS) (*n* = 11). Anthropometric measurements and body composition were obtained for each rower, and physical performance was measured by a 45-s supramaximal rowing test and a VO_2max_ incremental test on a Concept II rowing ergometer. The results showed that the rowers in the middle were taller (186.6 ± 4.9 cm), and significant differences were also found between the two groups according to body mass (BS 72.3 ± 3.8 vs. M 85.4 ± 4.3) and peak power (BS 641.5 ± 84 vs. M 737 ± 47.1), mean power (BS 538.5 ± 48.4 vs. M 604.1 ± 42.3), and physiological parameters (*p* < 0.05), VO_2max_ (BS 66.5 ± 4.9 vs. M 59.3 ± 6.7). It can be concluded that height could be associated with elite rowers’ performance and that a lower body mass index is related to better performance in bow and stern positions.

## 1. Introduction

Rowing is a cyclical endurance sport practiced worldwide [1], which comprises different disciplines: flat water rowing (e.g., Olympic rowing) and open water disciplines [2]. Within these open water modalities, Trainera (traditional rowing boat) regattas take place on the Cantabrian Sea in the north of Spain. These competitions involve completing a 5556 m (3 nautical miles) race on a Trainera crewed by thirteen rowers and a coxswain (known as Patrón) in the shortest possible time [3,4,5,6]. Unlike in Olympic rowing, there is no individual rowing, and it can be performed on the sea. Boat seats are fixed rather than mobile (as in Olympic rowing), which affects the speed of the boat, the total number of strokes, and the average force and power per stroke [2], thus making fixed seat rowing different.

Despite the existing differences between Olympic rowing and fixed seat varieties, there have been several publications that have compared them [2,7,8,9], because Olympic rowing is a benchmark for traditional rowing at all levels. Some studies related to training have been carried out on *Trainera* rowing [5,9,10,11,12] in order to better understand athletes’ physiological responses and, therefore, their performance. Although both Olympic and traditional rowing use the same primary energy system (glycolytic) [3,4,5,13], the physical demands for each of these sports are different. In *Trainera* regattas, it is estimated that a power of between 270 and 330 W is maintained during the 700–760 strokes, at a rate of 36–40 strokes per minute, in which the contribution of the lower body is close to 40% of the total power [5], with a significant demand being placed on the trunk musculature in fixed seat rowing [14]. Although the lactate concentrations reached by rowers depend on their position on the boat, concentrations between 10 and 18 mmol/l have been recorded [13,14], which enables them to race at a pace above the anaerobic threshold [8]. Different boat positions have also been defined in Olympic rowing, namely rowers in the middle of the boat and rowers in the bow and stern positions [15]. The main three boats in traditional rowing are called *Batel* (four rowers and a coxswain), Trainerilla (six rowers and a coxswain), and *Trainera* (thirteen rowers and a coxswain). The latter is considered the main type of boat [7]. Trainera rowing is performed all over the Cantabrian Sea in the north of Spain, and there are different leagues by competitive level and sex [2,16]. Around one hundred *Trainera* crews and over two thousand athletes currently compete during the summer period. There has been a growth in the number of participants in the last 10 years due to the presence of women’s and veterans’ competitions [17]. The elite male league is the Eusko Label Liga, organized by the Association of *Trainera* Clubs, known as ACT. It is composed by the best 12 clubs [2,16,18] and was created in 2003.

Sports performance has frequently been related to anthropometric parameters. This is the case for team sports such as volleyball, in which higher performance has been associated with the position played in, a low fat percentage, higher muscle mass, and height [19, 20], for individual sports such as swimming [21], cycling [22,23], judo [24], running [25,26,27,28], and also for lesser-known sports such as stand-up paddleboarding [29]. There is also a relationship between anthropometric variables and performance in Olympic rowing [30,31,32]. Data have shown that a taller rower with greater lean mass may have an advantage due to a longer lever arm, resulting in greater power output per stroke [33,34].

Although the scientific literature on traditional rowing is limited, it has also been stated that some anthropometric variables seem to play an important role in performance [2,35], suggesting that, while rowers with different anthropometric profiles are necessary in *Trainera* rowing, height and body mass correlate with male and female rowers’ performance [11,16,35,36]. However, León-Guereño, P. et al. [8] specified that height may not be as important for performance as wingspan and that a low body fat rate seems beneficial. It is worth noting that these studies have found a statistical relationship between the competitive level of rowers and their anthropometry [8] and between their anthropometry and performance in a 2 km race [35]. Moreover, recent research into *Trainera* rowing has shown anthropometric statistical differences among men and women rowers and with different levels of maximal aerobic power found in men [37]. Nevertheless, no studies have related anthropometric characteristics to the physiological responses of elite traditional rowers, nor has there been an analysis that considered boat positions. Therefore, considering this gap in the literature, the overarching objective was to investigate the anthropometry and performance variables of male elite *Trainera* rowers. The first aim of this research was to determine if the anthropometry, performance, and physiological capacities of traditional rowers are related to their position (in the boat’s extremities or the center) in the *Trainera*. The second aim was to determine the relationship between anthropometric variables of elite traditional rowers and physiological variables in anaerobic power/capacity and VO_2max_. The third aim was to study the relationships between different physiological variables among elite traditional rowers. It is hypothesized that the rowers in the central positions will be the ones with the greatest height, wingspan, and weight, and the ones capable of generating the most power.

## 2. Materials and Methods

### 2.1. Participants

Twenty elite male traditional rowers (29.4 ± 7) from the ACT (first division of the traditional rowing league) with experience of between 5 and 23 years volunteered for this study, these characteristics being inclusion criteria. All of them perform the same supervised training, 2–3 h a day, 7 days a week, although this varies depending on the season. Each rower received oral and written information about the objectives of the research, and all rowers gave written consent before participating. Failure to provide their consent in writing was an exclusion criterion. This study complied with the requirements of the Declaration of Helsinki and was approved by the Ethics Committee of the University of Deusto (ETK-13/18–19).

### 2.2. Procedure

**Figure 1:**
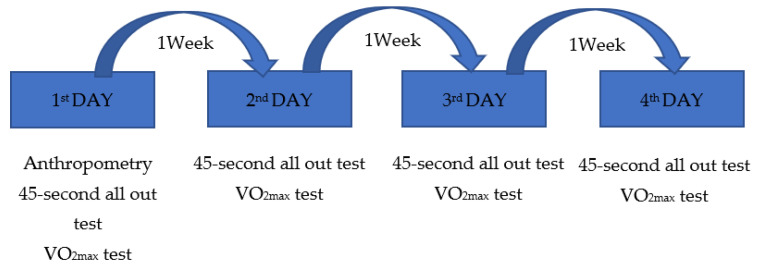
Weekly procedure and sequence of rowers’ data collection tests.

Rowers were tested for four consecutive weeks in the pre-competition period (Figure 1). To ensure full recovery, measurements were taken at one-week intervals. Participants were also asked not to perform any strenuous exercise 24–48 h before the evaluations and to eat a high-carbohydrate diet before the evaluation sessions. To avoid variations in performance due to changes in the time of day when the tests were conducted, all assessments were carried out at the same time of day.

On the first day, in addition to general or descriptive questions, an anthropometric study, a 45-s supramaximal test, and a test to determine VO_2max_ were performed. On the second, third and fourth days, the 45-s and VO_2max_ tests were repeated, in order to ensure greater reliability in the results.

The order of the physiological evaluations on the first day was as follows: a 45-s test and a VO_2max_ assessment were conducted with a 20-min rest between tests to ensure that lactate concentrations recovered to resting values. On the second, third, and fourth days, the 45-s test was conducted first in order to avoid interference between tests, followed by the VO_2max_ test. There was a resting period between the energy efficiency test and the VO_2max_ test of about 5 min, and between the initial 45-s test and the energy efficiency test of at least 20 min in order to ensure that the rowers returned to resting lactate concentrations.

Concept 2 Model C ergometers (Concept 2 Inc., Morrisville, VT, USA), which were calibrated according to the manufacturer’s recommendations, were used in all the assessments. The rowers were already familiar with these ergometers. A coupling was fitted to fix the seat to achieve a better simulation of the technical movement [10]; a drag factor of 160 was also utilized [38], since it was the drag factor value that showed the best agreement with fixed-seat rowing [39].

#### 2.2.1. Anthropometric Measurements

Height (in cm) was obtained using a SECA 220 measuring ruler (Hamburg, Germany) with an accuracy of 1 mm. Body mass (BM, in kg) was measured using Inbody 770 (USA) within 0.1 kg. Both measurements were taken with the subjects in their underwear. Height was monitored with the rowers standing upright and having their chins parallel to the ground. The body mass index (BMI) was calculated as body mass (kg) divided by height squared (m^2^).

All anthropometric measurements were performed according to the International Society for the Advancement of Kinanthropometry (ISAK) protocol (ISAK; 2016) by two international level 2 certified anthropometrists, respecting the corresponding intrapersonal technical error of measurement (TEM): 5% for skinfolds and 1% for the other measurements. All variables were measured on the right side of the body in duplicate, and the mean value was recorded.

Skinfolds (mm) (tricipital, bicipital, abdominal, suprailiac, subscapular, iliac crest, anterior thigh, and calf) were analyzed using a Holtain^®^ skinfold caliper with an accuracy of 0.2 mm. To obtain more information on body fat, the sums of 4 (∑4 SF), 6 (∑6 SF), and 8 (∑8 SF) skinfolds (mm) were examined by utilizing validated procedures (ISAK; 2016). Muscle perimeters (cm) (arm, contracted arm, thigh, waist, hip, and calf) were measured with a non-stretchable metal tape (Cercorf, Brazil) with an accuracy of 1 mm. The contracted arm and calf perimeters were corrected through skinfolds using the following formula [40] (Equation (1)):

Corrected perimeter = perimeter − (∏ × skinfold area)(1)

Bone length was measured from the proximal to the distal end of each using a Cerscorf anthropometer (Cerscorf, Brazil), with an accuracy of 1 mm. Fat mass (FM) and body fat percentage (BF%) were calculated by averaging the Carter, Faulkner, Yuhasz, and Withers equations following ISAK and the *Grupo Español de Cinantropometría* (GREC) recommendations for athletes [27,41]. The percentage of muscle mass (MM%) was calculated using the Lee equation [42], and the Carter and Heath equation was employed for somatotype values [43].

#### 2.2.2. The 45-s Supramaximal Test

Before completing the test, the subjects performed an 8-min warm-up at a perceived exertion intensity of 5–6/10 on the BORG scale [44] on a rowing ergometer with a drag factor of 160 [38,39].

This was a 45-s supramaximal test with verbal stimulus. The power output of each stroke measured in watt (W) was assessed by a computer integrated into the ergometer (Concept PM2), which provided the maximum (PP), mean (MP), and minimum (MinP) power recorded over 45 s. The fatigue index (FI) was then calculated [45] (Equation (2)):

FI = (PP − Minimum power)/PP) × 100(2)

#### 2.2.3. VO_2max_ Assessment

The VO_2max_ test was performed with an incremental ergometer test, starting at 135 W, increasing by 25 W every minute, and up to the level of voluntary exhaustion. The cadence for the rowers was free. Rowers were considered to have reached peak performance and, therefore, to have reached their VO_2max_ when at least two of the following criteria were met [46]: (i) a plateau in VO_2max_, defined as an increase of less than 1.5 mL-kg^−1^-min^−1^ in two consecutive workloads; (ii) a respiratory exchange ratio (RER) > 1.15; and (iii) a peak HR value (HR_max_) > 95% of the maximum predicted for age (220—age). Peak power output (PPO) (in W) was calculated as follows, taking into account each second (Equation (3)) [47]:

PPO = total completed intensity (W) + ((second at final speed/60 s) × 5 W)(3)

Exhaled gases were collected and analyzed using a calibrated continuous breath-by-breath gas exchange with the analyzer (Geratherm Respiratory Ergostik, Germany). The metabolic cart was calibrated according to the manufacturer’s recommendations before each test session.

### 2.3. Statistical Analyses

All data are expressed as mean ± SD. The Shapiro-Wilk normality test (<30) was performed to determine the normality of the variables examined. Levene’s test was conducted to establish the homoscedasticity of variances. The existence of outliers was determined, and no significant values were found. A one-factor ANOVA was used, taking boat position as a fixed factor to determine the differences between positions, anthropometry values, and physiological and physical performance. Partial eta squared (η^2^p) was used to calculate effect sizes across participants. As this measure could overestimate effect sizes, values were interpreted according to Ferguson [48].

Pearson’s bivariate correlation test was used to determine the correlation between anthropometric, body composition, and performance variables.

Statistical data analyses were performed using the Statistical Package for the Social Sciences 24.0 (SPSS, Inc., Chicago, IL, USA). The statistical significance for all analyses was set at *p* < 0.05.

## 3. Results

As far as differences between positions are concerned (Table 1), significant differences (*p* < 0.05) were found between rowers in positions at the boat’s extremities (bow and stern) and rowers in the boat’s central positions (3rd and 4th). The rowers in central positions had greater height, weight, sitting height, wingspan, and lean mass values than those at the bow and stern.

On the other hand, bow/stern rowers had significantly lower subscapular and abdominal skinfolds than 3rd–4th rowers, plus the sum of 8 skinfolds (*p* < 0.05) (Table 2). In addition, the bow/stern rowers had a significantly larger head, neck, relaxed arm, corrected arm, contracted arm, wrist, mesosternum, waist, and thigh (1 cm larger than 3rd–4th rowers) (*p* < 0.05).

Finally, bow/stern rowers had significantly shorter acromion-radiale, mid stylion-dactylion, trochanterion-tibiale, foot, and tibiale mediale-sphyrion than 3rd–4th rowers (*p* < 0.05).

Regarding performance, both VO_2max_ and variables related to anaerobic performance, power max, and mean in the 45-s test are significantly different between both groups of rowers (Table 3).

Regarding the basic anthropometric measures of the rowers and their correlation with the different physiological variables (Table 4), height, weight, sitting height, and wingspan were correlated with performance in average power and maximal power in the anaerobic test. Likewise, Fat Avg Equations and MM were correlated with some performance data.

Some of the lengths and perimeters of the rowers were also highly related to differences in performance (Table 5). All measured lengths except the radiale-stylion had an influence on 45-s anaerobic test performance (both peak and average power). As far as perimeters are concerned, practically half of the perimeters were related to the VO_2max_ in the performance test (head, neck, relaxed arm, waist, mesosternum, mid-thigh…). This ¡ measurement was one of the most closely related to anthropometric variables in general (skinfolds and perimeters).

Finally, Table 6 shows how, in addition to the logical relationship between different variables of the same test (Power_ Med_45s and Power_ Max_45s for example), a relationship was found between all the powers recorded in the 45-s test (Max, Med, and Min) and the Power Max and the VO_2max_ by the rowers.

## 4. Discussion

The objectives of this research were, firstly, to determine if there were differences in anthropometry, performance, and the physiological capacities of traditional rowers depending on their boat positions; and secondly, to discover the relationship between the anthropometric variables of elite rowers and the physiological variables in power/anaerobic capacity and VO_2max_. The rowers in the 3rd and 4th central positions had significant differences in terms of height, weight, wingspan, and seated height and had a higher MM%, PP, and MP in the 45-s test. Height, weight, wingspan, sitting height, and other anthropometric variables such as some lengths were related to higher performance in PP and MP in the 45-s test, higher VO_2max_ and power at VO_2max_.

The results obtained in this research are partially in line with previous studies carried out in rowing, since a significant relationship between anthropometry variables and performance was found [37]. Metrics like height, weight, body mass, and wingspan have been shown to be correlated with rowing performance in both Olympic rowing [49,50] and traditional rowing [8,15], thus showing the importance of these variables in their relationship to sport performance in this specific sport [37]. Moreover, it is known that in *Trainera* rowing, rowers of smaller stature and body weight are needed for certain positions, especially in the bow and stern, so the crews formed are not as uniform as in Olympic rowing boats [7]. This means that the ideal morphology of all rowers in traditional rowing is not similar, unlike in Olympic rowing. Due to the needs of each position in the *Trainera* in terms of physical and technical/tactical demands, it has been observed that those crew members who row in the central positions in the boat (3rd–4th) preferably have significantly larger bodies in terms of height, weight, BMI (body mass index), wingspan, sitting height, and MM. As far as somatotype is concerned, less endomorphic and more mesomorphic values were recorded than those reported by León-Guereño et al. [8], but no significant differences were found between the different positions. These results could make sense when associated with the characteristics of this type of rowing, since it is practiced at sea, and with the characteristics of the boat, which is wider in the center and makes it easier for larger rowers to row in the most efficient way possible without a hydrodynamic penalty.

Similarly, this study showed that the rowers in the third and fourth positions were the tallest and heaviest and had significantly higher values in some of the most important physiological variables recorded, such as PP and MP. These results are partially in line with a previous study on Mediterranean traditional rowing, which showed that height, body mass, and body musculature correlated with rowing performance in male and female rowers. Similarly, propulsive speed, average power, and peak power were correlated with athletes’ performance [51]. As in Olympic rowers, it may seem logical that elite rowers who are taller and heavier, have a higher MM, or greater wingspan, would report better performance [30,49], due to their greater capacity for force production by larger levers and power generation, among other things [52]. These characteristics appear to be consistent across different categories and ages of elite rowers. Internationally ranked rowers exhibited significantly greater body height, body mass, sitting height, arm length, limb length, and body surface area. Additionally, they row 2000 m significantly faster and have higher values for power, relative power, jump height, maximum speed, and maximum strength [32]. These anthropometric characteristics have also been associated with long-term career attainment in elite junior rowers [53] and are therefore related to talent identification and programs. However, in *Trainera* rowing, it is the central positions that allow for this type of rower due to the characteristics of the boat, as both the stern and bow positions are narrower, and at the same time, the weight in the center of the boat will make navigation easier; therefore, rowers equivalent to light rowers in Olympic rowing will be necessary for the bow and stern positions.

Our results are partially consistent with Akça [31], who predicted rowing ergometer performance from functional anaerobic power, strength, and anthropometry in Olympic rowing. This author measured anaerobic power by an “all out” 30-s effort, which was related to athletes’ performance in the 2000 m test and their anthropometric characteristics [31], in line with the results obtained in our research, with a significant relationship of height, weight, and BMI with performance variables like 45-s “all out” and VO_2max_. This correlation between certain anthropometric variables and performance variables was consistent with previous studies on Olympic rowing [54] and probably could be explained by the relationship between different morphologies and performance variables [43]. However, these results should be treated with caution, since the findings obtained here according to different positions in *Trainera* rowing might lead us to define an ideal anthropometric model for different positions within the vessel.

The main limitation of our study was that it was limited to only twenty rowers, eleven in the bow and stern positions, and nine in the middle of the boat. Future investigations should be carried out with a larger sample size from the different positions on the boat and also including women who are *Trainera* rowers [37]. However, this investigation was the first attempt to better understand the relationship between anthropometric characteristics and performance variables according to the positions in *Traineras*, following investigations carried out in other sports [20]. Moreover, since the sample consisted of elite athletes in this rowing discipline, the results obtained should be given due consideration.

## 5. Conclusions

In conclusion, this investigation showed that there are significant differences between the various boat positions regarding anthropometric characteristics and performance, and that rowers in the middle of the boat showed higher values in height, weight, wingspan, seated height, MM%, PP, and MP in the 45-s test. Moreover, athletes’ performance in PP and MP in the 45-s test, VO_2max_, or power at VO_2max_, was related to variables such as their height, weight, wingspan, sitting height, and other anthropometric variables such as some lengths. These findings could be relevant for coxswains to help them better plan and adapt rowers’ training sessions, which could vary even depending on their boat position. Moreover, a better understanding of performance-related anthropometric variables could also help optimize athletes’ performance, help coxswains identify talent [53], and determine the type of rowers that should be in a *Trainera*.

## Figures and Tables

**Table 1:** Rowers’ basic anthropometric measures and body composition data based on their boat position.

	Bow/Stern (n = 11)		3rd–4th (n = 9)			
	Mean (SD)	Range (Min–Max)	Mean (SD)	Range (Min–Max)	*p*	η^2^p
Height (cm)	177.5 (3.8)	170.2–183.3	186.6 (4.9)	176.6–191.5	<0.001 *	0.544
Weight (kg)	72.3 (3.8)	65.9–77.8	85.4 (4.3)	79.3–93.0	<0.001 *	0.749
BMI	22.9 (1.3)	21.4–25.7	24.6 (1.3)	23.3–26.9	0.013 *	0.300
Seated size (cm)	142.3 (2.1)	138.3–145.7	146 (2.1)	142.2–149.2	0.001 *	0.449
Wingspan (cm)	180.8 (5.1)	171.5–187.5	186.8 (5.5)	179.0–197.5	0.017 *	0.276
Avg. body fat formulas (%)	9.1 (1.7)	7.2–13.1	12 (4.1)	8.0–20.2	0.045	0.204
MM Lee (Kg)	35.1 (1.4)	32.64–36.8	39.3 (1.7)	36.3–42.2	<0.001 *	0.666
MM Lee (%)	48.8 (1.2)	46.6–50.5	46.1 (1.5)	43.3–47.8	<0.001 *	0.519
Endomorphy	2.1 (0.5)	1.5–3.3	2.8 (1.2)	1.5–5.3	0.162	0.127
Mesomorphy	5.1 (0.7)	4.0–6.3	5.2 (0.5)	4.6–6.2	0.879	0.001
Ectomorphy	2.6 (0.7)	1.2–3.5	2.5 (0.7)	1.1–3.1	0.576	0.018

*p*: significant differences between groups by one-factor ANOVA. BMI = body mass index; * = Statistical Significance *p* < 0.05; Sum 8 = sum of 8 body folds; % Avg. body fat Formulas = fat% from the average results of Carter, Whiters, Faulkner, and Yuhasz equations; MM% Lee = % muscle mass using Lee’s equation.

**Table 2:** Rowers’ skinfolds, perimeters, and lengths based on their boat position.

	**Bow/Stern** (**n = 11**)		**3rd–4th** (**n = 9**)			
	**Mean** (**SD**)	**Range** (**Min–Max**)	**Mean** (**SD**)	**Range** (**Min–Max**)	***p***	**η^2^p**
Skinfolds (mm)						
Triceps	7.9 (2.7)	4.3–13.9	9.0 (4.1)	4.5–15.4	0.469	0.029
Subscapular	8.1 (1.4)	6.8–11.8	11.7 (4.6)	8.4–23.4	0.024	0.250
Biceps	3.8 (1.5)	2.3–7.9	4.6 (1.9)	2.5–8.3	0.306	0.058
Iliac Crest	10.4 (3.7)	6.5–19.6	18.1 (8.8)	8.8–34.1	0.016	0.282
Supraspinal	6.9 (1.8)	5.1–11.0	10.2 (5.3)	5.6–22.4	0.071	0.170
Abdominal	10.9 (3.9)	7.0–18.8	21.9 (11.5)	10.3–43.1	0.008	0.334
Thigh	10.9 (4.6)	5.7–19.0	12.8 (5.7)	6.4–21.8	0.425	0.036
Calf	6.2 (2.5)	3.8–11.2	8.0 (4.7)	3.8–16.6	0.278	0.065
Sum 8	65 (18.4)	44.7–110.4	96.2 (43)	53.6–178.6	0.042	0.210
Perimeter (cm)						
Head	56.2 (1.2)	54.4–58.5	58.3 (1.6)	55.7–60.1	0.003	0.387
Neck	35.9 (1.1)	34.1–37.9	37.9 (1.2)	35.7–39.5	0.001	0.464
Relaxed arm	30.5 (1.3)	28.3–32.2	33.0 (1.4)	30.5–35.2	0.001	0.475
Corrected arm	29.7 (1.5)	26.9–31.6	32.1 (1.1)	30.1–33.7	0.001	0.464
Contracted arm	32.9 (1.5)	31.0–34.9	35.2 (1.1)	33.5–37.1	0.001	0.446
Forearm	27.9 (0.7)	27.0–29.0	28.5 (2.0)	23.3–30.2	0.394	0.041
Wrist	16.6 (0.4)	15.8–17.1	18.8 (3.2)	17.2–27.2	0.029	0.237
Mesosternum	97.8 (2.5)	94.5–101.1	104.2 (3.0)	99.7–109.6	<0.001	0.610
Waist	77.2 (2.9)	72.6–82.2	87.0 (4.8)	81.1–96.4	<0.001	0.642
Hip	89.4 (18.3)	35.4–101.5	100.8 (3.2)	95.9–106.3	0.082	0.159
Waist Hip ratio	0.94 (0.41)	0.78–2.18	0.86 (0.03)	0.81–0.91	0.592	0.016
Thigh 1 cm	56.0 (2.5)	52.1–59.7	59.4 (2.9)	56.0–64.6	0.012	0.300
Medium thigh	53.6 (2.6)	50.3–57.9	55.9 (2.6)	51.3–59.7	0.063	0.179
Corrected medium thigh	52.5 (2.8)	48.8–56.6	54.7 (2.4)	50.2–57.7	0.089	0.152
Calf	36.7 (1.4)	34.7–39.3	37.3 (1.6)	35.1–39.3	0.632	0.046
Corrected calf	36.1 (1.4)	34.2–38.8	36.5 (1.5)	34.5–38.7	0.508	0.025
Ankle	22.4 (1.2)	20.7–24.3	23.1 (0.8)	22.1–24.6	0.155	0.109
Length (cm)						
Acromion-Radiale	32.2 (1.4)	28.8–34.7	33.8 (1.2)	31.9–35.9	0.014	0.290
Radiale-Stylion	25.8 (2.0)	23.2–29.7	27.1 (1.0)	25.1–28.4	0.094	0.148
Mid Stylion-Dactylion	19.2 (0.5)	18.3–19.7	19.8 (0.8)	19.0–21.6	0.028	0.241
Trochanterion-Tibiale	36.4 (2.6)	33.3–40.8	40.1 (3.2)	35.2–44.3	0.011	0.312
Foot	26.8 (0.6)	26.0–27.6	28.1 (1.2)	25.9–30.2	0.004	0.369
Tibiale mediale-Sphyrion	37.9 (1.1)	35.5–39.2	40.7 (2.0)	37.9–44.3	0.001	0.472

*p*: significant differences between groups by one-factor ANOVA. Sum 8 = sum of 8 body skinfolds.

**Table 3:** Rowers’ performance data based on their boat position.

	Bow/Stern (n = 11)		3°–4° (n = 9)			
	Mean (SD)	Range (Min–Max)	Mean (SD)	Range (Min–Max)	*p*	η^2^p
VO_2max_	66.5 (4.9)	57.7–72.9	59.3 (6.7)	47.8–70.2	0.012 *	0.302
Power at VO_2max_	309.1 (25.2)	285.0–360.0	326.7 (21.6)	285.0–360.0	0.116	0.132
PP 45 s	641.5 (84)	553.0–799.0	737 (47.1)	642.0–797.0	0.007 *	0.338
Mean P 45 s	538.5 (48.4)	492.0–611.0	604.1 (42.3)	544.0–662.0	0.005 *	0.360
Min P 45 s	465.9 (24.9)	427.0–498.0	497.1 (56.7)	421.0–575.0	0.117	0.131
FI 45 s	26.6 (7.6)	11.2–40.4	32.5 (6.8)	24.05–41.78	0.085	0.156

*p*: significant differences between groups by one-factor ANOVA. * = Statistical Significance *p* < 0.05; PP 45 s = peak power 45 s; Mean P 45 s = mean power 45 s; Min P 45 s = minimum power 45 s; FI 45 s = fatigue index 45 s.

**Table 4:** Correlation between basic anthropometric measures and body composition and performance data.

	VO_2max_	Power at VO_2max_	PP 45 s	MP 45 s	Min P 45 s	FI 45 s
Height	−0.405	0.073	0.404	0.546 *	0.232	0.464 *
Weight	−0.686 **	0.032 *	0.544 *	0.592 **	0.342	0.386
BMI	−0.619 **	0.182	0.406	0.320	0.285	0.067
Seated size	−0.438	0.247	0.238	0.509 *	0.152	0.480 *
Wingspan	−0.159	0.076	0.543*	0.620 **	0.331	0.419
Fat Avg Equations	−0.786 **	0.803	0.054	0.991	0.208	0.107
MM Kg	−0.617 **	0.059	0.555*	0.600 **	0.331	0.442
MM% Lee	0.603 **	0.040 *	−0.399	−0.426	−0.290	−0.174
Endomorphy	−0.748 **	0.708	0.016	−0.017	−0.214	0.218
Mesomorphy	0.001	0.630	0.213	0.142	0.271	−0.138
Ectomorphy	0.368	0.693	−0.207	−0.045	−0.162	0.137

Data are expressed by Pearson’s r. * *p* < 0.05; ** *p* < 0.01. MM: muscle mass using Lee’s equation.

**Table 5:** Pearson’s correlation between skinfolds, perimeters, lengths, and performance variables.

	VO_2max_	Power at VO_2max_	PP 45 s	MP 45 s	Min P 45 s	FI 45 s
Skinfolds						
Triceps	−0.629 **	−0.175	−0.173	−0.223	−0.314	0.085
Subscapular	−0.670 **	0.158	0.237	0.228	−0.061	0.329
Biceps	−0.668 **	−0.084	0.016	−0.067	−0.237	0.233
Iliac Crest	−0.851 **	0.069	0.158	0.122	−0.082	0.283
Supraspinal	−0.767 **	0.163	0.128	0.071	−0.191	0.312
Abdominal	−0.827 **	0.235	0.239	0.229	0.027	0.278
Thigh	−0.481 *	−0.334	−0.272	−0.310	−0.473 *	0.090
Calf fold	−0.622 **	−0.135	−0.219	−0.206	−0.305	0.011
Sum 8	−0.801 **	0.032	0.062	0.031	−0.181	0.243
**Perimeters**						
Head	−0.802 **	0.254	0.312	0.316	0.276	0.161
Neck	−0.691 **	0.375	0.383	0.435	0.359	0.143
Relaxed arm	−0.541 *	0.280	0.360	0.412	0.228	0.249
Corrected arm	−0.441	0.323	0.406	0.469 *	0.296	0.242
Contracted arm	−0.409	0.186	0.420	0.448 *	0.171	0.364
Forearm	−0.442	−0.087	0.120	0.046	0.021	0.133
Wrist	−0.349	0.270	0.411	0.490 *	0.594 **	0.001
Mesosternum	0.005 *	0.279	0.050 *	0.045 *	0.354	0.114
Waist	−0.705 **	0.429	0.503 *	0.528 *	0.236	0.394
Hip	−0.424	0.209	−0.189	−0.026	0.088	−0.251
Waist Hip Ratio	0.171	−0.037	0.390	0.217	0.003	0.404
Thigh 1 cm	−0.856 **	0.511 *	0.280	0.273	0.223	0.134
Medium thigh	−0.593 **	0.573 **	0.285	0.312	0.279	0.076
Corrected thigh	−0.507 *	0.636 **	0.336	0.370	0.366	0.060
Calf	−0.331	0.539 *	0.368	0.352	0.343	0.114
Corrected calf	−0.183	0.593 **	0.437	0.418	0.434	0.115
Ankle	−0.288	0.571 **	0.458 *	0.429	0.292	0.265
**Lengths**						
Acromion-Radiale	−0.287	0.424	0.500 *	0.443	0.179	0.421
Radiale-Stylion	0.066	0.234	0.352	0.359	0.165	0.292
Stylion mid dactylion	−0.098	0.606 **	0.493 *	0.634 **	0.483 *	0.157
Trochanterion-Tibiale	−0.417	0.131	0.460 *	0.488 *	0.232	0.358
Foot	−0.118	0.680 **	0.619 **	0.684 **	0.515 *	0.271
Tibiale mediale-Sphyrion	−0.348	0.624 **	0.615 **	0.608 **	0.284	0.467 *

Data are expressed by Pearson’s r. * *p* < 0.05; ** *p* < 0.01. PP 45 s = peak power 45 s; MP 45 s = mean power 45 s; Min P 45 s = minimum power 45 s; FI 45 s = fatigue index 45 s; Sum 8 = sum of 8 body folds. P = perimeter; L = length.

**Table 6:** Pearson’s correlation between different physiological parameters.

	VO_2max_	Power at VO_2max_	PP 45 s	MP 45 s	Min P 45 s	FI 45 s
VO_2max_	-	−0.095	−0.042	−0.053	−0.013	−0.054
Power at VO_2max_	-	-	0.582 **	0.661 **	0.657 **	0.114
PP 45 s	-	-	-	0.939 **	0.535 *	0.717 **
MP 45 s	-	-	-	-	0.731 **	0.489 *
Min P 45 s	-	-	-	-	-	0.405
FI 45 s	-	-	-	-	-	-

Data are expressed by Pearson’s r. * *p* < 0.05; ** *p* < 0.01. PP 45 s = peak power 45 s; MP 45 s = mean power 45 s; Min P 45 s = minimum power 45 s; FI 45 s = fatigue index 45 s.

## Data Availability

The raw data supporting the conclusions of this article will be made available by the authors without undue reservation.
